# In-Plane Mechanical Behavior Design of a Locally Rib-Reinforced Rotating Hexagonal Honeycomb

**DOI:** 10.3390/biomimetics11030172

**Published:** 2026-03-02

**Authors:** Jialiang Xie, Jinjin Huang, Xiaolin Deng

**Affiliations:** 1 School of Mechanical and Electrical Engineering, Guilin University of Electronic Technology, Guilin 541004, China; xiejialiang_2020@163.com (J.X.); huangjinjin0104@163.com (J.H.); 2 School of Mechanical and Resource Engineering, Wuzhou University, Wuzhou 543003, China

**Keywords:** rotated structure, rib-reinforced, energy absorption, dynamic response

## Abstract

This study develops a novel Locally Rib-Reinforced Rotational Hexagonal Honeycomb (LRRH) model. The objective is to systematically enhance the model’s mechanical performance and energy absorption efficiency through geometric morphology construction. The structure combines triangular and hexagonal units through a rotational arrangement, forming a rotating rigid structure (RRH), upon which re-entrant parallelogram units are embedded. A Finite Element simulation was developed in Abaqus/Explicit. Its reliability was validated by comparing the numerical predictions against the outcomes of quasi-static compression experiments. The axial impact response and energy absorption attributes of the configuration were thoroughly evaluated by adjusting the hexagonal cell angles and applying a symmetric design approach. The experimental outcomes indicate that the SEA of the RRH-Type I-180°-180° model surpasses that of the RRH-Type I-105°-105° by 43.68%, and the SEA of the LRRH-Type I-105°-105° achieved a significant 97.88% increase compared to the LRRH-Type I-180°-180° variant. Meanwhile, the SEA of the RRH-Type I-180°-180° honeycomb increased by 121.2% and 11.79% compared with the LRRH-Type I-180°-180° and LRRH-Type I-105°-105° structures. Parametric analysis results indicate that wall thickness and impact velocity are critical factors influencing energy absorption performance. The enhancement of structural thickness considerably strengthens its flexural resistance and pressure tolerance.

## 1. Introduction

Following the emergence of the lightweight design principles, honeycomb architectures have been widely adopted across a number of engineering domains, including aerospace, transportation, civil engineering, energy systems, and biomedical implants. This widespread adoption is attributable to their low weight, high strength, exceptional energy absorption capacity, and remarkable efficiency in space utilization [1,2]. Recently, honeycomb architectures exhibiting negative Poisson’s ratio (Auxetic) effects [3,4,5] have garnered increasing research interest. These structures demonstrate unconventional mechanical behavior, featuring lateral expansion under tension and lateral contraction under compression, which starkly contrasts with the behavior exhibited by traditional positive Poisson’s ratio honeycombs [6]. Their distinctive deformation mechanisms and superior overall performance provide considerable advantages in applications such as impact protection, energy absorption, and controllable deformation systems [7]. Representative examples of auxetic honeycomb configurations encompass re-entrant hexagonal [8,9], arrowhead-shaped [10,11], star-shaped [12,13], and chiral honeycombs [14,15]. These structures achieve the negative Poisson effect through innovative geometric design, significantly enriching both structural design strategies and the engineering application scenarios of auxetic materials. They exhibit exceptional performance in terms of shear stiffness [16,17,18], impact capability [19,20,21], and energy absorption capacity [22,23]. Their suitability for the design of multifunctional, lightweight, and high-performance architectures offers extensive development prospects and considerable research significance.

With the increasing demand for lightweight designs and enhanced energy absorption performance, the optimization of honeycomb structures has attracted considerable attention. In recent years, various honeycomb configurations based on triangular [24,25] and hexagonal [26,27] units have been developed, making the investigation of their structural mechanics and energy absorption characteristics a core focus in this field [28,29,30]. Nedoushan et al. [31] put forth a completely triangular honeycomb structure featuring an anti-tetrachiral configuration. A systematic investigation revealed that the structure demonstrates a consistent auxetic effect in both axial and lateral orthogonal directions, in addition to possessing a relatively high specific stiffness. Zhang et al. [32] put forth a self-similar hierarchical triangular honeycomb structure. Drawing on experimental findings and finite element simulations, the study demonstrated that this structure exhibits significant improvements in compressive performance, deformation patterns, and energy absorption capacity when set against conventional triangular honeycombs. Zhang et al. [33] introduced triangular lattices at the vertices of conventional hexagonal honeycombs to construct a novel vertex-based fractal honeycomb structure and investigated its mechanical response under both in-plane and transverse compression. The findings revealed that the fractal design significantly altered the failure modes of the honeycomb, with notably superior performance observed under in-plane compression. Xue et al. [34] proposed a rotational triangular auxetic metamaterial, achieving lightweight design and mechanical performance optimization through a perforated configuration. The findings from quasi-static compression tests and numerical simulations revealed that the rotational triangular design achieved a four-time increase in plateau stress, along with a tenfold enhancement in specific energy absorption. Ding et al. [35] put forth a multilayer perforated rotating triangular auxetic metamaterial, incorporating secondary circular or triangular perforations to achieve lightweight design. The results demonstrated that these designs can significantly enhance the specific energy absorption while preserving the elastic modulus, strength, and auxetic characteristics. Wei et al. [13] systematically investigated and assessed the mechanical response of star-shaped triangular honeycomb structures under quasi-static in-plane compression. The findings demonstrated that the star-shaped triangular honeycomb possesses a sustained and substantial negative Poisson’s ratio characteristic.

Beyond refining the geometry of honeycomb structures, the incorporation or reintegration of supporting ribs or other structural elements within the honeycomb cells has also proven to be a viable strategy for boosting mechanical performance [7,36,37]. Zhang et al. [38] proposed an auxetic metamaterial composed of wedge-shaped re-entrant units. The introduction of wedge-like components enabled stiffness modulation during compression and suppressed lateral buckling, thus enhancing structural stability and the prominence and consistency of the auxetic effect. Experimental and numerical simulation results demonstrated that this structure possesses excellent stiffness tunability and adjustable auxetic behavior. Zou et al. [39] introduced arched ribs into re-entrant hexagonal structures. Through the synergistic effect of structural reinforcement and auxetic deformation, the energy absorption performance was enhanced. The findings suggested that the arched ribs could stabilize and regulate the deformation process, and the stress–strain curves exhibited two distinct yield plateaus. Shi et al. [40] proposed a novel aluminum honeycomb structure by embedding periodically dense S-shaped and I-shaped ribs within conventional hexagonal aluminum honeycomb cells. The results indicated that the locally densified structure outperforms traditional honeycombs in terms of energy absorption, specific energy absorption, mean crushing force, and crushing efficiency. Liu et al. [41] designed a novel hourglass-shaped honeycomb structure based on the concave hexagonal honeycomb configuration and developed four enhanced variants by embedding transverse or longitudinal stiffening ribs. A finite element analysis model was employed to systematically investigate the impact resistance and energy absorption performance of these structures under varying impact velocities and geometric parameters. Lai et al. [42] introduced rib reinforcements into re-entrant honeycomb structures and developed a two-dimensional equivalent homogenized model based on the energy method to systematically investigate their static and dynamic performance. Parametric analysis indicated that the rib thickness has a limited effect on structural performance, while an equilateral triangular arrangement tends to reduce the overall performance of the structure.

Building upon the aforementioned studies, this work combines triangle–hexagon composite honeycomb structures with local ribbed supports and refined unit design to boost the overall load-bearing strength and energy absorption performance. Within this framework, a novel Locally rib-reinforced Rotational Hexagonal Honeycomb structure is proposed. The structure uses rotationally arranged hexagons as the primary framework, embeds triangular units within, and incorporates ribs inside the triangles to form a parallelogram support system. The rib arrangement effectively disperses local stresses within the units, improving the overall stiffness and deformation control of the honeycomb structure. Furthermore, through a rotated and staggered unit arrangement strategy, the geometric connections between adjacent units are optimized, ensuring uniform distribution of local supports while maintaining the continuity of the honeycomb topology. This design effectively improves load transfer paths and mitigates stress concentration. Combined with experimental verification and numerical simulations, this study systematically evaluates the mechanical performance and energy absorption characteristics of the RRH honeycomb structure. The proposed design not only establishes a novel and effective pathway for optimizing high-performance honeycomb structures but also offers theoretical guidance and practical design insights for their application in engineering practice.

## 2. Structural Design

**Figure 1:**
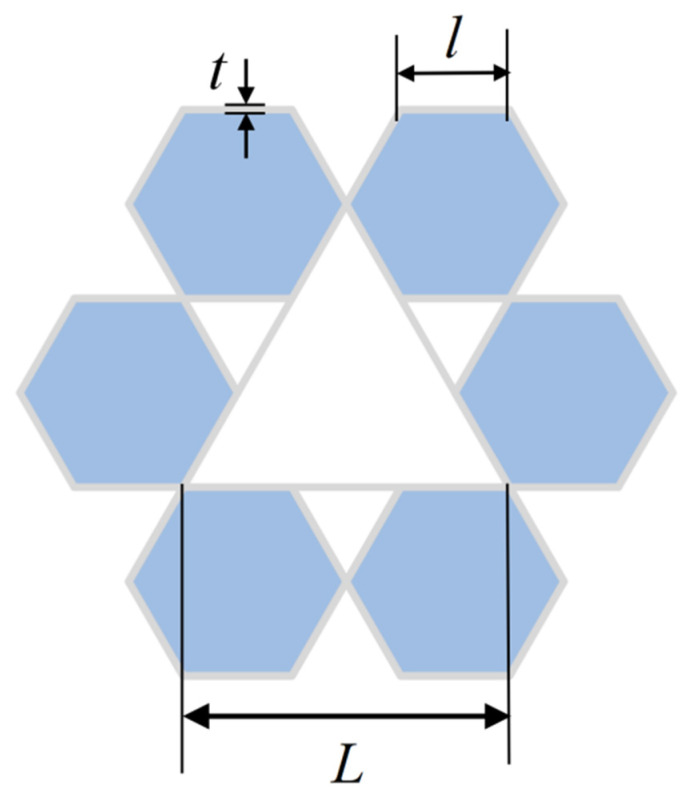
Rotationally rigid hexagonal design.

**Figure 2:**
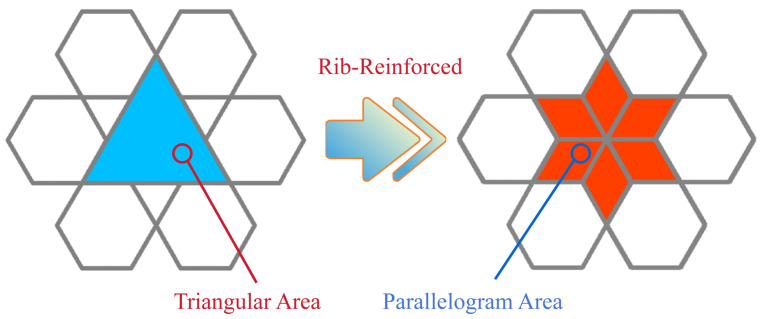
Transition process of the rib-reinforced structure.

As depicted in Figure 1, the proposed design features a rotating rigid hexagonal honeycomb structure. This configuration comprises six regular hexagonal cells arranged in a rotationally symmetric manner around a central triangular point and interconnected via composite hinge joints. This design fully exploits the inherent stability of the triangular units and their inward contraction-induced negative Poisson’s ratio effect, induced by inward contraction, to enhance the structure’s adaptability under load. Meanwhile, the regular hexagonal units exhibit excellent energy absorption characteristics, and their combination with the triangular units synergistically elevates the overall impact resilience of the honeycomb structure. In engineering design, parallelogram units, owing to their excellent geometric adaptability and stable kinematic constraint relationships, enable controllable displacement transmission and structural reconfiguration. Based on this concept, a rib-reinforcement design strategy is adopted, in which ribs are introduced within triangular units and the original structure is subdivided into multiple parallelogram sub-units, thereby forming a multi-level structural system with a coordinated deformation mechanism (as depicted in Figure 2). This design aims to optimize internal load-transfer paths, enhance local constraint effects, and suppress non-coordinated instability, ultimately improving the overall stability and load-bearing capacity of the honeycomb structure. Key geometric parameters of the honeycomb include the side length L of the equilateral triangle formed by the hexagonal array, the side length l of each regular hexagon, and the wall thickness t.

**Figure 3:**
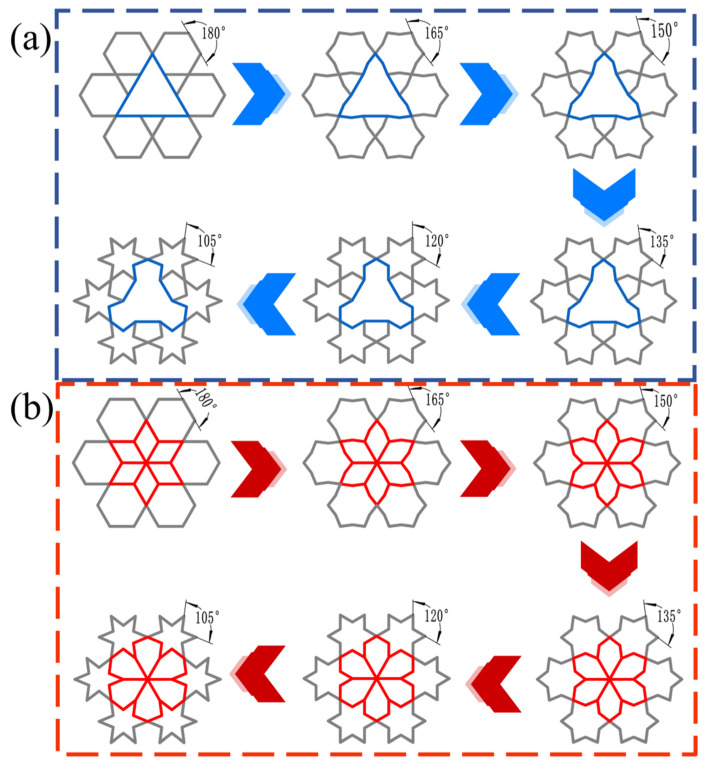
Schematic diagram of subunit size and evolution. (**a**) RRH; (**b**) LRRH.

As illustrated in Figure 3, a series of subunit configurations with varying internal angles was generated by applying a concave transformation to the regular hexagonal geometry. These included structures designated as RRH-Type I-180°-180°, RRH-Type I-165°-165°, RRH-Type I-150°-150°, RRH-Type I-120°-120°, and RRH-Type I-105°-105°. For clarity, a naming convention “RRH-Type Z-α°-β°” is employed, where Z denotes the structural type of the honeycomb configuration, α° represents the internal angle of the bottom subunit, and β° corresponds to that of the top subunit. For instance, RRH-Type I-180°-180° refers to a Type I honeycomb structure composed of subunits with internal angles of 180°, as depicted in Figure 2.

## 3. Materials and Methods

### 3.1. Finite Element Model

**Figure 4:**
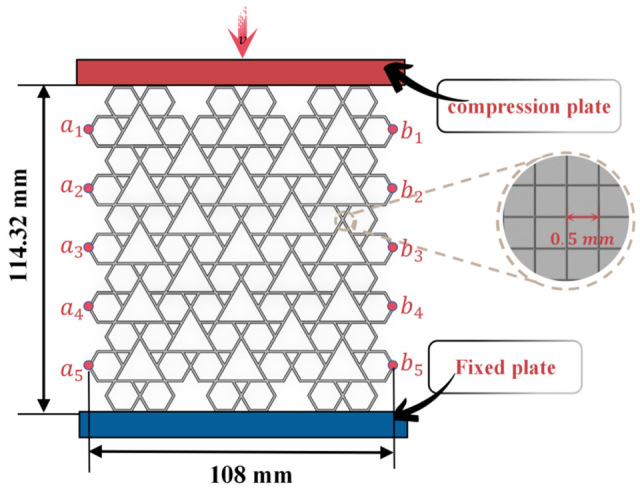
Finite element model development.

Numerical simulations were performed in Abaqus/Explicit based on the developed finite element model. The finite element impact model is illustrated in Figure 4. For the RRH-type structure, the key geometric parameters were as follows: cell angle of 180°, L = 18 mm, l = 6 mm, height = 114.32 mm, width = 12 mm, and wall thickness t = 0.8 mm. Rigid plates were incorporated at both the upper and lower boundaries of the model, with analytical rigid body definitions employed to represent the indenter and bottom platform used in physical experiments. The honeycomb structure was meshed using S4R shell elements [43]. The lower plate was rigidly attached to the honeycomb model, with all degrees of freedom completely constrained, thereby prohibiting any displacement or rotation in all directions (Ux = Uz = Uy = 0). The upper plate was allowed to translate solely in the vertical direction, with lateral displacements restricted (Ux = Uz = 0). A vertical impact was applied to the top plate at a uniform velocity of 10 m/s [44,45]. The impact displacement was established set to 80% of the honeycomb height (H = 114.32 mm), equivalent to 91.456 mm. The honeycomb model was discretized employing four-node reduced integration shell elements, featuring five integration points along the loading velocity direction to ensure numerical convergence. Two reference points, indicated in red and located on the left and right sides of the honeycomb, were designated to monitor horizontal displacement during compression. To preclude interpenetration between the honeycomb structure and the rigid plates, surface-to-surface contact was established between the honeycomb and the top and bottom rigid plates, employing a single-sided automatic contact algorithm. A friction coefficient of 0.2 [46,47] was assigned for tangential behavior, while normal contact behavior was modeled using a general contact definition to guarantee accurate and stable contact interactions. The honeycomb structure is composed of aluminum alloy AA6061-O, characterized by a Young’s modulus E = 70 GPa, density ρ = 2700 kg/m^3^, Poisson’s ratio v = 0.3, and yield stress = 130 MPa.

### 3.2. Mesh Division Test

**Figure 5:**
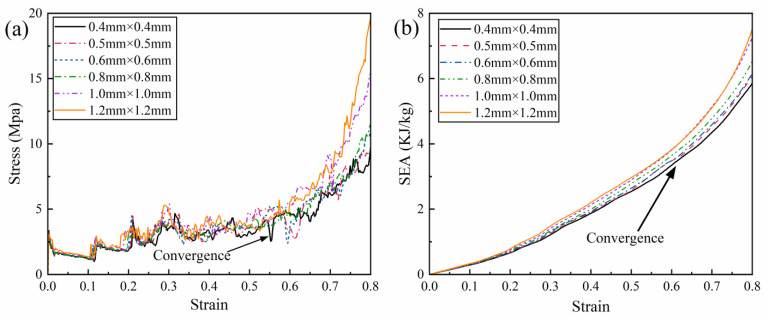
Mesh sensitivity results: (**a**) stress–strain curve; (**b**) SEA curve.

In light of the potential for mesh size to affect the reliability of numerical simulation results, this study performed a mesh convergence evaluation on the RRH-Type I-180°-180° honeycomb structure, with a wall thickness of t = 0.8 mm. The evaluation spanned six varying mesh sizes: 0.4 mm, 0.5 mm, 0.6 mm, 0.8 mm, 1.0 mm, and 1.2 mm. The results of these tests are visualized in Figure 5.

Standard metrics for evaluating the performance of honeycomb structures encompass macroscopic stress and macroscopic strain, which are defined as follows [48]:

(1) $$\sigma =\frac{F}{Lb}$$

(2) $$\varepsilon =\frac{∆H}{H}$$

In these equations, F stands for the force applied during the impact process, while L and b refer to the width and out-of-plane depth of the RRH, respectively. Moreover, ΔH indicates the impact distance, and H characterizes the initial height of the honeycomb structure.

Specific energy absorption (SEA) denotes the energy dissipated per unit mass, and its calculation formula is expressed as follows [22,49]:

(3) $$SEA=\frac{EA}{m}=\frac{v_{t}\int_{0}^{\varepsilon_{d}}\sigma (\varepsilon )d\varepsilon }{m}$$

In this formula, m refers to the total mass of the honeycomb, and V signifies the honeycomb’s overall volume.

As demonstrated in Figure 5, mesh size markedly influences the stress response and energy absorption behavior of the honeycomb structure. From Figure 5a, it is clearly observed that the peak stresses for the 0.4 mm and 0.5 mm mesh sizes are closely aligned, with the nominal stress–strain curves exhibiting a high degree of concordance. Furthermore, Figure 5b reveals that the energy absorption curves for these two mesh sizes nearly coincide. To strike a balance between computational cost and simulation accuracy, a mesh size of 0.5 mm was eventually chosen for subsequent analyses. This size guarantees reliable computational precision while significantly lowering the computational expense, thus establishing a robust basis for further model investigations.

### 3.3. Finite Element Analysis Model Validation

**Figure 6:**
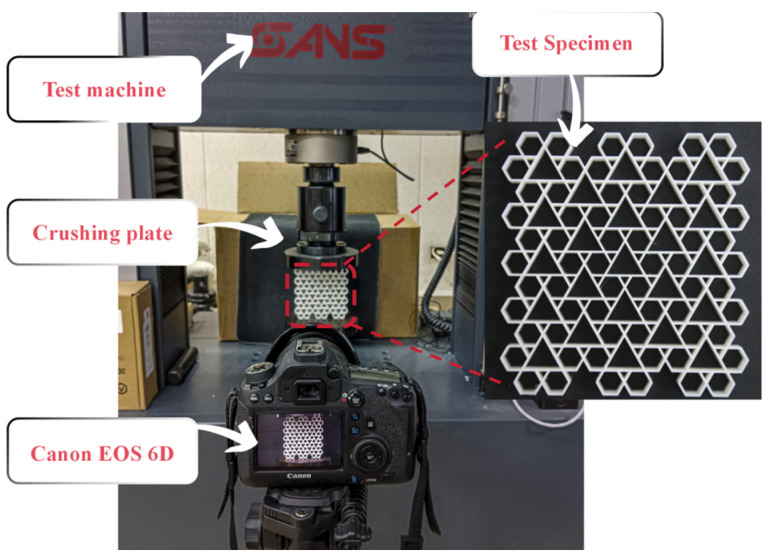
Quasi-static compression test.

**Figure 7:**
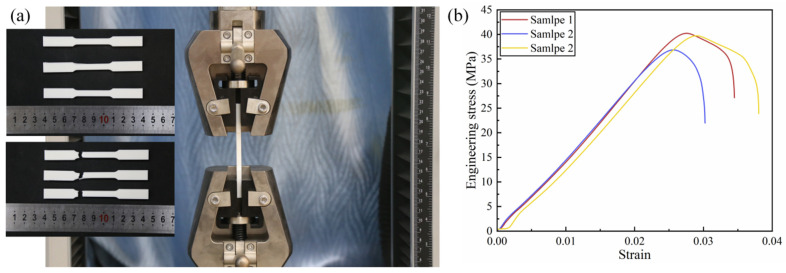
Tensile test and material properties: (**a**) tensile test, (**b**) stress–strain curve of PLA material.

This section details the validation of the finite element model through quasi-static compression tests in Figure 6. The RRH-Type I-180°-180° honeycomb structures and tensile specimens were fabricated using a RAISE 3D Pro Plus 3D printer (Shanghai UnionTech Co., Ltd., Shanghai, China), employing polylactic acid (PLA) as the printing material. The printing parameters were established with a nozzle temperature of 230 °C, a bed temperature of 60 °C, and a nozzle diameter of 0.4 mm. In order to ascertain the constitutive parameters of the PLA material, two tensile specimens were prepared according to the ASTM E8M standard. The honeycomb specimens possessed dimensions of 114.32 mm in height, 12 mm in in-plane tensile width, and a wall thickness of 0.8 mm. Tensile tests were conducted on a CMT6104 electronic universal testing apparatus, yielding the PLA’s engineering stress–strain characteristics and essential material parameters, all of which are displayed in Figure 7. Subsequently, quasi-static compression measurements were executed on the RRH honeycomb samples utilizing a CMT5205 universal testing machine. The samples were fastened onto a horizontal baseplate and compressed vertically by the upper platen at a loading rate of 2 mm/min [30,34]. The experimental arrangement is displayed in Figure 6. To ensure data stability, a preloading procedure was executed prior to formal loading. Throughout the compression process, the testing apparatus continuously logged load and displacement data in real time using a computer-controlled acquisition system. Additionally, a Canon 6D camera was employed to dynamically capture and document the deformation behavior of the honeycomb structure during the compression process. In the finite element simulations, a loading speed of 1 m/s [34,50] was adopted to replicate quasi-static compression conditions while minimizing the influence of inertial effects. This methodology enhances computational efficiency and ensures consistency between the simulation results and experimental observations. Table 1 lists the material properties of PLA.

**Figure 8:**
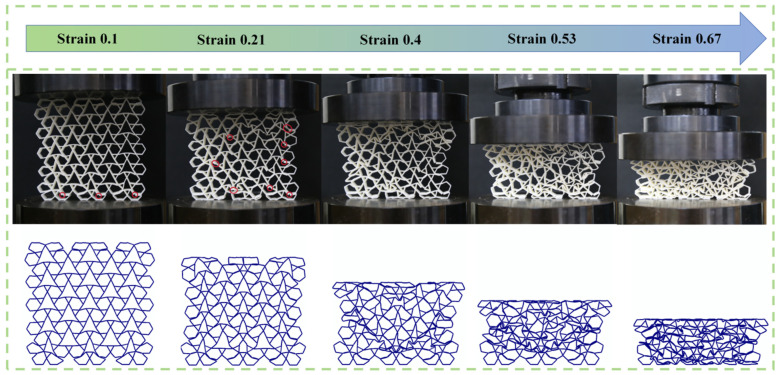
Comparison of experimental and simulation deformation modes.

**Figure 9:**
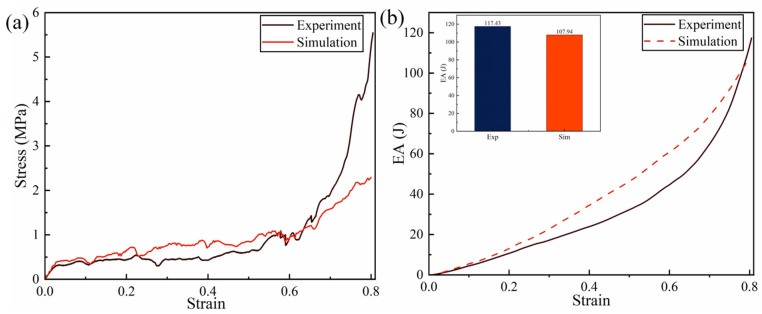
Comparison of experimental and simulation results: (**a**) stress–strain curves; (**b**) energy absorption curves.

The experimental results, as shown in the deformation comparison between experiment and simulation in Figure 8, clearly indicate the occurrence of local fractures in the early stage of compression. This phenomenon is primarily due to the inherent brittleness of the PLA material, which lacks sufficient toughness and is prone to brittle failure in regions experiencing stress concentration. Such fractures lead to significant local deformation. However, the overall deformation pattern remains highly consistent with the simulation results. Quantitative comparison between experimental and simulation results, as presented in Figure 9, indicates that the stress–strain curves in Figure 9a demonstrate favorable concurrence during the initial phase of compression. However, as compression progresses, the overall stress values in the experimental curve are marginally lower than those in the simulation. This discrepancy is primarily attributed to the formation of local fractures during the experiment, which reduces the structural load-bearing capacity, thereby decreasing the overall mechanical response. In contrast, the finite element model does not account for material fracture behavior, consequently failing to capture the observed stress degradation. The energy absorption curves illustrated in Figure 9b indicate some discrepancies between the experiment and simulation during the intermediate compression phase, manifesting as a relatively lower rate of increase for the experimental curve. This is attributed to localized excessive bending resulting from fractures, which reduces the energy absorption capacity of the honeycomb cells. Nonetheless, the final total energy absorption difference between experiment and simulation is only 8.8%, which suggests a strong convergence between the two sets of results. In summary, although the brittleness of the PLA material induces local failures during the experiment, leading to certain deviations in deformation patterns and mechanical performance, this impact is overall controllable and does not alter the primary deformation mechanisms. The finite element model has demonstrated high reliability in characterizing the mechanical response of the honeycomb structure, making it an effective tool for subsequent parameter studies and structural improvement.

## 4. Results and Discussion

### 4.1. Effect of Different Angles

#### 4.1.1. RRH Deformation Mode

**Figure 10:**
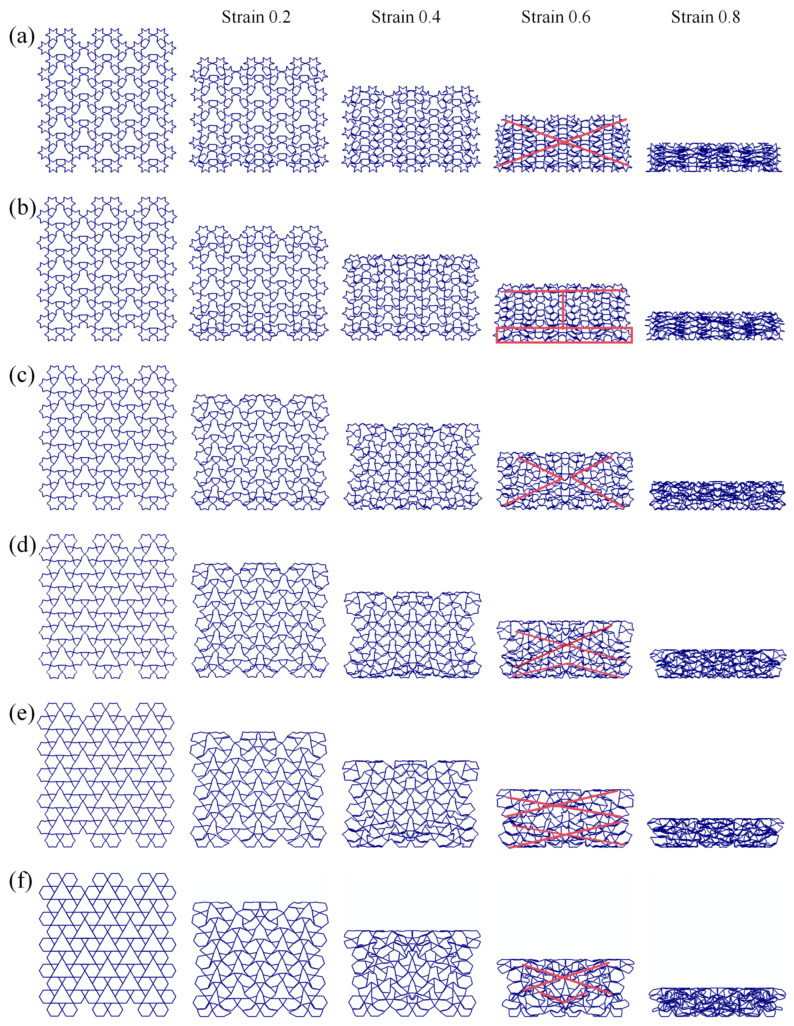
Deformation modes of RRH-Type I: (**a**) RRH-Type I-105°-105°, (**b**) RRH-Type I-120°-120°, (**c**) RRH-Type I-135°-135°, (**d**) RRH-Type I-150°-150°, (**e**) RRH-Type I-165°-165°, and (**f**) RRH-Type I-180°-180°.

Figure 10 illustrates the geometric schematics and deformation mechanisms of the RRH-Type I honeycomb structures. When the nominal strain reaches 0.6, the RRH-Type I-105°-105° honeycomb exhibits collapse in an “X”-shaped pattern; the RRH-Type I-120°-120° honeycomb demonstrates an overall “工”-shaped deformation mode prior to crushing; the RRH-Type I-135°-135° honeycomb displays “V”-shaped deformation patterns towards both the left and right sides, followed by inward contraction and collapse; the RRH-Type I-150°-150° honeycomb reveals an “X”-shaped deformation at the top and a “V”-shaped compression at the bottom; the RRH-Type I-165°-165° honeycomb shows “X”-shaped deformation modes at both the top and bottom, compressing and contracting inward; finally, the RRH-Type I-180°-180° honeycomb exhibits an “X”-shaped deformation in the mid-upper region and a “V”-shaped deformation at the bottom, leading to overall inward contraction and crushing. Analysis of these deformation patterns indicates a significant alteration in the deformation mode of the RRH-Type I series honeycombs as the concave angle increases. The mode transitions from predominantly “X” and “工” shapes to a combination of “X”-shaped deformation at the top and “V”-shaped deformation at the bottom. This behavior is attributed to the evolution of the hexagram-like structures within the honeycomb from regular hexagons. This phenomenon can be attributed to the geometric reconfiguration of the honeycomb cells and the evolution of their load transfer paths under compression. When the concave angle is small, the cell exhibits a star-shaped (hexagram) configuration, where approximately linear struts form a stable load-bearing network. Axial loads are distributed along multiple paths, effectively suppressing local buckling in the central region and maintaining high overall stability during the initial compression stage. As the concave angle increases, the cell geometry gradually transitions toward a regular hexagonal topology, weakening the original oblique load paths, reducing lateral constraints, and inducing a pronounced inward contraction.

**Figure 11:**
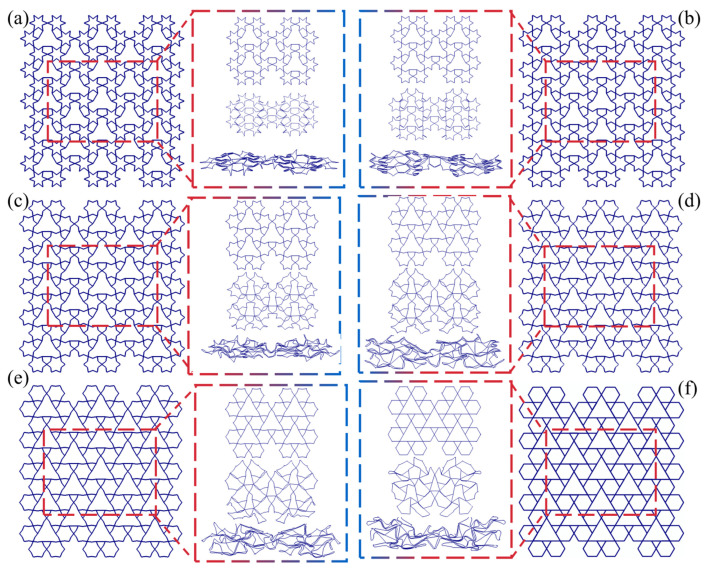
Comparison of subunit deformation processes for different structures: (**a**) RRH-Type I-105°-105°, (**b**) RRH-Type I-120°-120°, (**c**) RRH-Type I-135°-135°, (**d**) RRH-Type I-150°-150°, (**e**) RRH-Type I-165°-165°, and (**f**) RRH-Type I-180°-180°.

Figure 11 illustrates the five central subunit structures of the RRH-Type I honeycomb and their corresponding deformation modes. As depicted, the deformation behavior of these subunits under axial loading is significantly influenced by the concave angle. When the concave angle is relatively small, the subunits exhibit stable deformation during compression, and the overall structure shows no significant inward contraction. However, as the concave angle increases, the honeycomb begins to exhibit pronounced inward contraction under axial impact. This contraction suppresses the bending and rotational deformation of the outer rib regions, thereby limiting their capacity to effectively resist compressive loads.

#### 4.1.2. LRRH Deformation Mode

**Figure 12:**
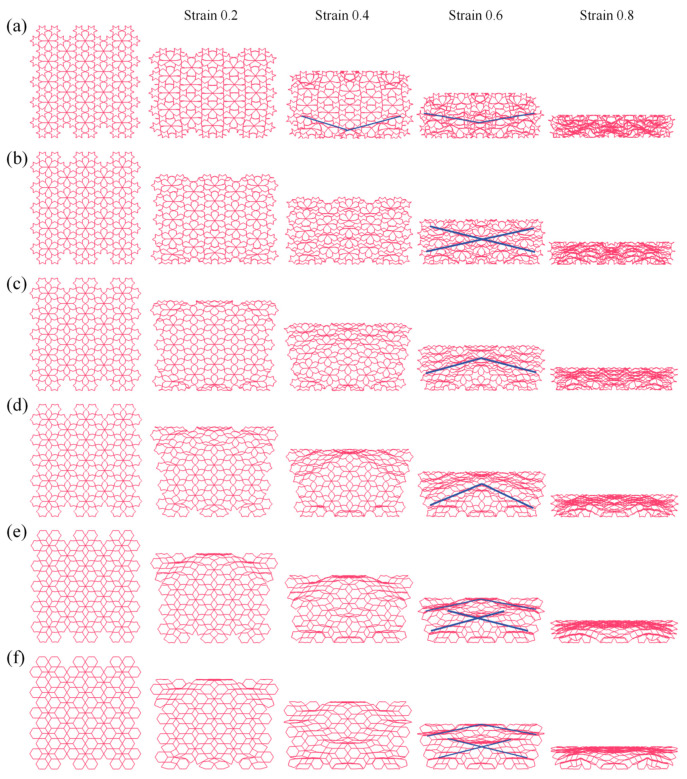
Deformation modes of LRRH-Type I structures: (**a**) LRRH-Type I-105°-105°, (**b**) LRRH-Type I-120°-120°, (**c**) LRRH-Type I-135°-135°, (**d**) LRRH-Type I-150°-150°, (**e**) LRRH-Type I-165°-165°, and (**f**) LRRH-Type I-180°-180°.

Figure 12 presents the schematic diagram and typical deformation patterns of the LRRH-Type I honeycomb structures. The analysis indicates that the LRRH-Type I-105°-105° honeycomb begins to demonstrate a “V”-shaped deformation at the bottom when the nominal strain reaches 0.4. As the strain further increases to 0.6, the entire structure collapses in a “V”-shaped manner. The LRRH-Type I-120°-120° honeycomb exhibits an “X”-shaped deformation pattern at the onset of compression. Both the LRRH-Type I-135°-135° and LRRH-Type I-150°-150° honeycombs collapse in a downward “V”-shaped deformation. Meanwhile, the LRRH-Type I-165°-165° and LRRH-Type I-180°-180° configurations demonstrate a combined deformation mode characterized by a downward “V”-shaped deformation at the top and an “X”-shaped pattern at the bottom. These deformation modes suggest that when the concave angle is relatively small, the honeycomb structures tend to deform in “V” or “X” patterns. This phenomenon is mainly ascribed to the initial distortion of the outer ribs under axial load, which progressively shifts a portion of the load to the inner supporting ribs, culminating in the structure’s global deformation. Throughout this process, the synergistic interaction between the outer ribs and the internal supporting ribs effectively distributes the applied load, thereby ensuring structural stability and promoting a more uniform densification of the honeycomb. However, as the concave angle increases, the deformation mode undergoes a significant transformation, shifting to a combined pattern characterized by a downward “V”-shaped deformation at the top and an “X”-shaped deformation at the bottom. This transition is fundamentally attributed to the reduced axial load-bearing capacity of the outer ribs in honeycombs with larger concave angles, resulting in a higher load concentration on the internal supporting ribs. The underlying cause of this transition lies in the reduced axial load-bearing capacity of the outer ribs in honeycombs with large concave angles, which leads to a concentration of stress on the internal supporting ribs. As a result, these ribs undergo more pronounced bending and compressive deformation, exacerbating the overall structural deformation. At a nominal strain of 0.8, noticeable gaps remain at the base of the honeycomb, indicating that full densification has not yet been achieved. This observation suggests that honeycombs with larger concave angles exhibit limited compressive performance and cannot reach optimal densification at relatively low strain levels.

**Figure 13:**
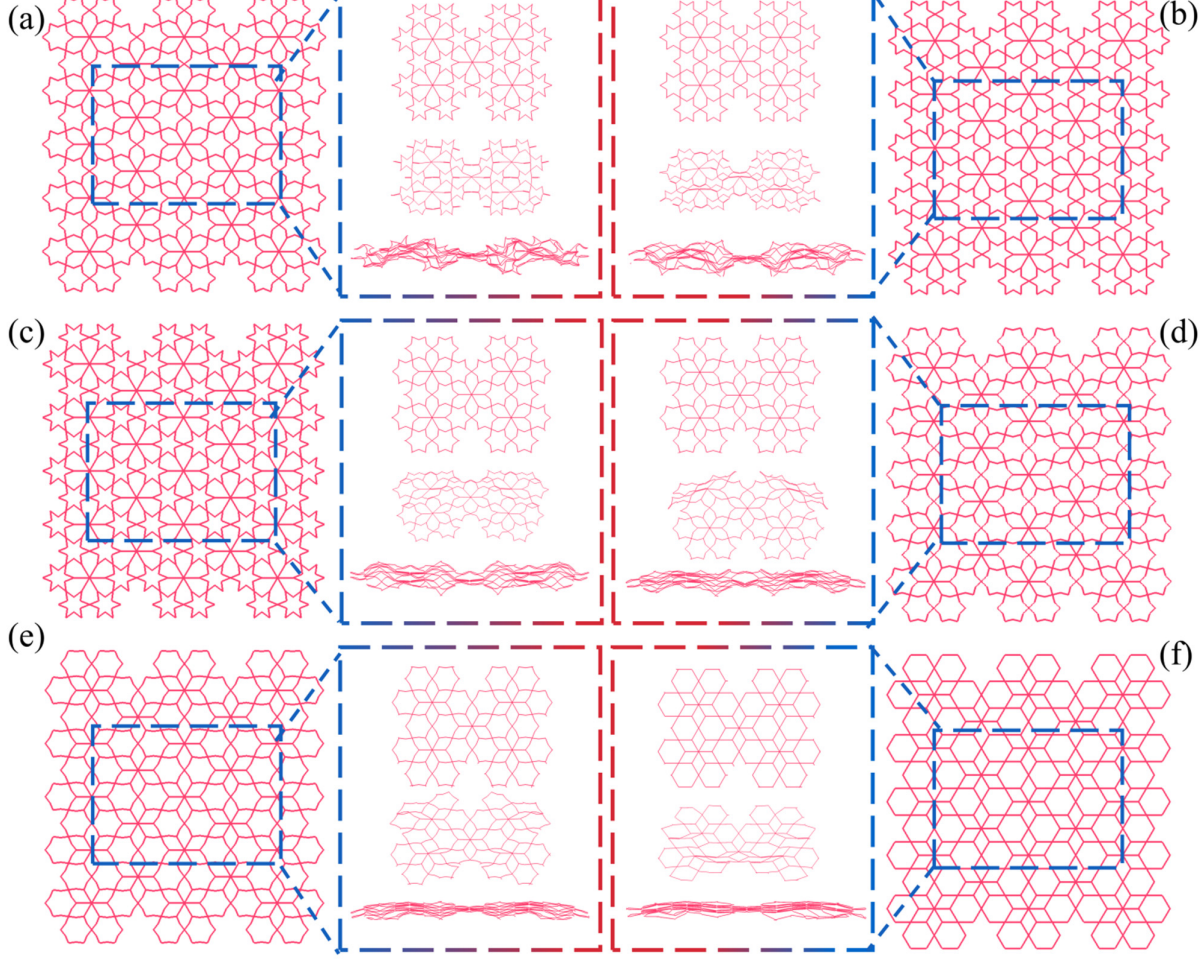
Comparison of subunit deformation processes for different structures: (**a**) LRRH-Type I-105°-105°, (**b**) LRRH-Type I-120°-120°, (**c**) LRRH-Type I-135°-135°, (**d**) LRRH-Type I-150°-150°, (**e**) LRRH-Type I-165°-165°, and (**f**) LRRH-Type I-180°-180°.

Figure 13 illustrates the middle five subunits of the LRRH-Type I honeycomb structure and their corresponding deformation modes. The deformation pattern of the subunits in the LRRH-Type I honeycomb is closely associated with the concave angle. As depicted, when the concave angle is relatively small, both the outer ribs and the internal supporting ribs undergo coordinated deformation and contraction. The load is transmitted from the outer ribs to the supporting ribs, resulting in relatively uniform structural compression. Conversely, at larger concave angles, the axial compressive resistance of the outer ribs gradually diminishes, leading to an increasing concentration of load on the internal supporting ribs. Consequently, the supporting ribs exhibit preferential deformation, characterized by significant compression and bending behavior.

#### 4.1.3. Impact Resistance Comparison

**Figure 14:**
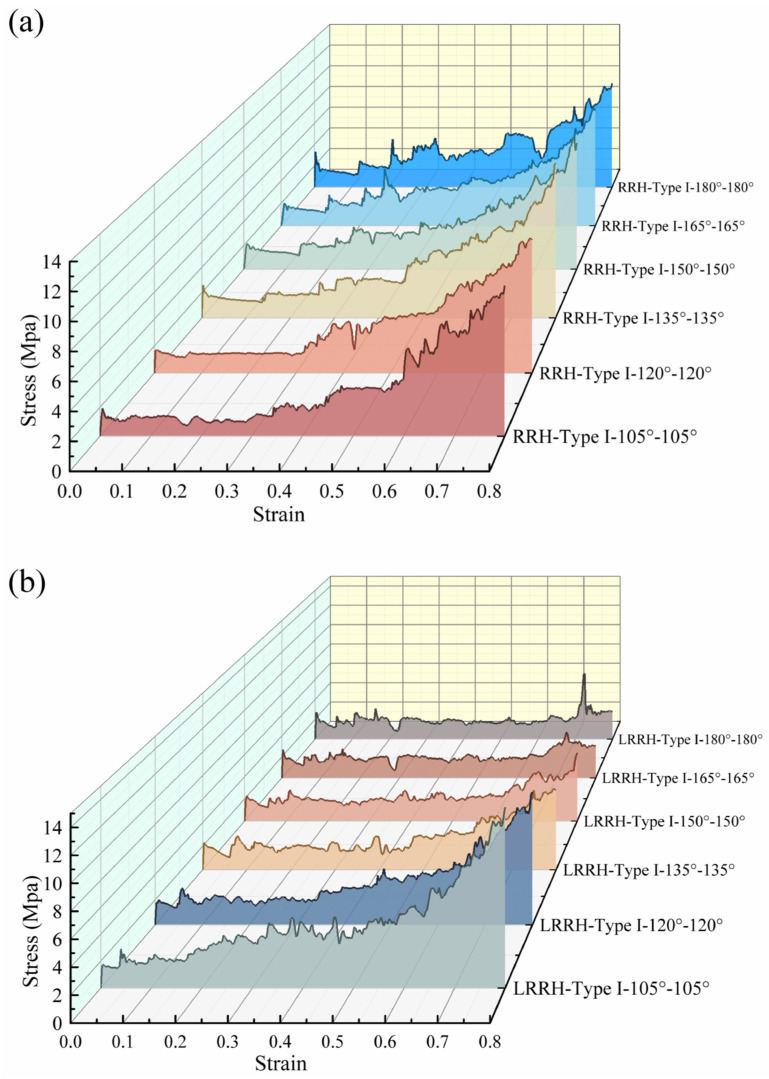
Stress–strain curves: (**a**) RRH-Type I; (**b**) LRRH-Type I.

**Figure 15:**
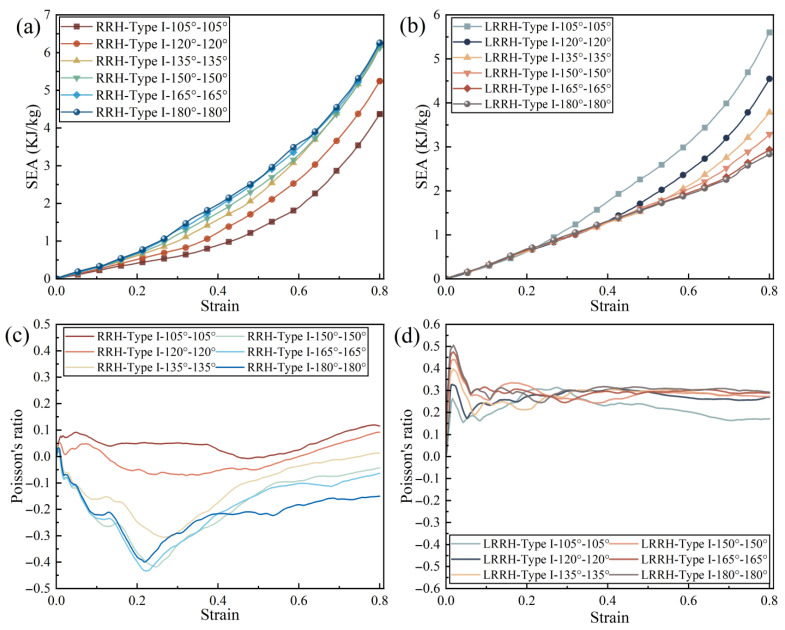
Specific energy absorption and dynamic Poisson’s ratio curves: (**a**) RRH-Type I; (**b**) LRRH-Type I; (**c**) RRH-Type I; (**d**) LRRH-Type I.

Figure 14 and Figure 15 present the stress–strain curves, the SEA–strain curves, and the dynamic Poisson’s ratio–strain curves, respectively. As illustrated in Figure 4, ten points were selected from the structure to extract displacements in both the X and Y directions for the calculation of the dynamic Poisson’s ratio. The dynamic Poisson’s ratio is computed using the following equations:

(4) $$∆\bar{L}=\frac{1}{5}\sum_{i=1}^{5}\left(L_{bi}-L_{ai}\right)$$

(5) vd=−εxεy=∆L¯L∆yH

In this context, ${\;L}_{\mathrm{ai}}$ and $L_{\mathrm{bi}}\;$ denote the displacements of points *a* and *b*, respectively. (see Figure 3) $∆\overset{-}{L}\;$ denotes the average displacement in the X-direction, and ∆y indicates the corresponding displacement in the Y-direction. The dynamic Poisson’s ratio $v_{d}$ is calculated based on Equation (5).

To reduce subjective bias, the densification strain was determined using the energy absorption efficiency method, which is defined as [1]:

(6) $$\frac{dE(\varepsilon )}{d\varepsilon }{|}_{\varepsilon =\varepsilon_{d}}=0$$

Among them, E is the energy absorption efficiency ratio parameter of the honeycomb. This energy efficiency parameter can be calculated by the following formula [51]:

(7) $$E=\frac{\int_{0}^{\varepsilon }\sigma (\varepsilon )d\varepsilon }{\sigma (\varepsilon )}\;$$

$\sigma_{m\;}$ denotes the average nominal stress during the impact process, which is expressed as follows:

(8) $$\sigma_{m}=\frac{1}{\varepsilon_{d}}\int_{0}^{\varepsilon_{d}}\sigma (\varepsilon )d(\varepsilon )$$

In the equation, σ(ε) is the nominal stress corresponding to the nominal strain ε, and $\varepsilon_{d}$ denotes the densification strain.

**Figure 16:**
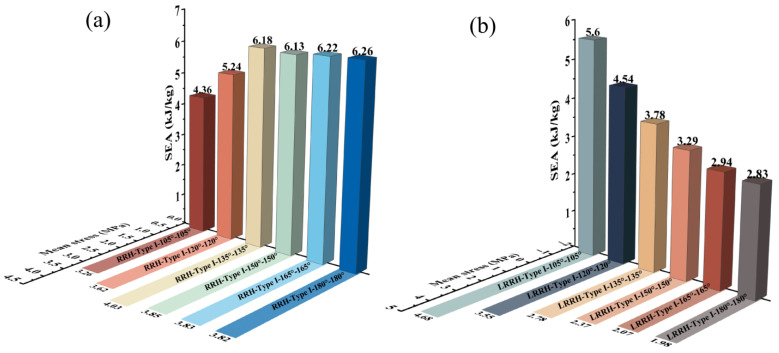
Performance comparison of different structures: (**a**) RRH-Type I; (**b**) LRRH-Type I.

From Figure 14a, as the re-entrant angle increases, both the initial maximum stress and the overall plateau stress of the RRH-Type I honeycomb structures display a progressive rising tendency. This phenomenon suggests that a larger concave angle diminishes the resistance of the outer ribs to axial loads, resulting in the honeycomb structure progressively contracting inward during compression. Concurrently, the folding direction of the internal triangular support ribs gradually aligns with the compression direction, causing them to bear more stress and undergo greater localized deformation during loading, thereby effectively enhancing the structure’s capacity to withstand axial impact. Notably, the stress–strain curves reveal three distinct plateau stages, indicating that the honeycomb structure undergoes a stepwise evolution during compression, characterized by initial local collapse followed by overall deformation. As shown in Figure 15a, the SEA curve of the RRH-Type I-180°-180° honeycomb structure consistently exceeds that of the RRH-Type I structure, indicating that the former possesses superior energy absorption capability throughout the compression process. However, during the compression process, the SEA curves of the RRH-Type I-135°-135°, RRH-Type I-150°-150°, and RRH-Type I-165°-165° honeycomb structures gradually converge toward that of the RRH-Type I-180°-180° honeycomb. Figure 15c presents the dynamic Poisson’s ratio curves, which reveal that the negative Poisson’s ratio effect progressively strengthens with the increase in the concave angle. When the concave angle reaches 165°, the negative Poisson’s ratio effect attains its maximum value, indicating that at this angle, the lateral expansion of the honeycomb structure is significantly enhanced, resulting in a more pronounced negative Poisson’s ratio behavior during axial compression. As shown in Figure 16a, the RRH-Type I-135°-135° honeycomb exhibits the highest value, while the values for RRH-Type I-150°-150°, RRH-Type I-165°-165°, and RRH-Type I-180°-180° honeycombs are closely aligned. This observation suggests that during the densification stage, the internal triangular support ribs undergo nearly complete bending deformation, and the honeycomb structure begins to collapse as a whole, causing their energy absorption mechanisms to converge. Overall, in comparison to the RRH-Type I-105°-105° honeycomb, the RRH-Type I-180°-180° honeycomb demonstrates a 43.68% increase in SEA and a 25.83% increase in $\sigma_{m}$. This suggests that the deformation process of the RRH-Type I-180°-180°honeycomb exhibits notable superiority regarding energy dissipation and load-carrying capacity. It effectively utilizes the internal support ribs and structural configuration of the honeycomb, enhancing its compressive strength and energy dissipation capability. Consequently, the RRH-Type I-180°-180° honeycomb exhibits superior mechanical performance compared to other concave-angle honeycomb structures, particularly in energy absorption and load-bearing capacity, underscoring its substantial research value.

Figure 14b explicitly displays the stress–strain characteristics curves of the LRRH-Type I honeycomb structures. As the concave angle increases, the initial maximum stress shows a progressive upward trend, while the plateau stress undergoes a notable reduction. This behavior, in conjunction with the deformation process of the honeycomb, suggests that larger concave angles increasingly limit the compressive capacity of the honeycomb, thus preventing it from achieving maximum densification at lower strains. Specifically, larger concave angles result in a more stable and slower deformation of the honeycomb structure, thereby restricting the densification rate during the initial stage. This prolongs the duration of the plateau phase during deformation, inhibiting a rapid transition to a complete collapse state, which ultimately leads to a gradually reduced shaded area enclosed by the curve and the *x*-axis. As depicted in Figure 15b, the SEA curve for the LRRH-Type I-105°-105° honeycomb consistently surpasses that of the LRRH-Type I honeycomb. This phenomenon arises because at larger concave angles, the energy absorption capacity of the honeycomb does not increase rapidly during the initial stage; rather, it exhibits an extended stable compression phase. Although the initial peak stress is elevated, the gradual decline in plateau stress indicates that energy absorption efficiency diminishes during the later stages. Figure 15d presents the Poisson’s ratio curves of the LRRH-Type I honeycomb structures. From this figure, it is evident that the LRRH-Type I structure consistently demonstrates a positive Poisson’s ratio throughout the compression process. This behavior indicates that under compressive loading, the lateral strain of the honeycomb structure is positively correlated with the longitudinal strain. A positive Poisson’s ratio typically suggests relatively uniform deformation, primarily dominated by local bending and buckling of the honeycomb walls. Further analysis of Figure 16b reveals that as the concave angle continues to increase, both the SEA and $\sigma_{m}$ average stress exhibits a pronounced decline, characterized by a sharp “cliff-like” drop. This indicates that excessively large concave angles diminish the overall deformation resistance and energy absorption performance of the honeycomb structure. In comparison, the LRRH-Type I-105°-105° honeycomb shows a 97.88% increase in SEA and a 127.04% increase in average stress $\sigma_{m}$ relative to the LRRH-Type I-180°-180°, thereby highlighting its significant advantages in energy absorption capacity and load-bearing performance.

Based on the above results, it can be observed that, with increasing re-entrant angles, the energy absorption capacity of the RRH-Type I honeycomb structure gradually increases, whereas that of the LRRH-Type I honeycomb decreases. Meanwhile, the negative Poisson’s ratio effect of the RRH honeycomb strengthens with increasing re-entrant angle, while the LRRH exhibits a positive Poisson’s ratio effect. This difference is primarily attributed to the internal deformation mechanisms: during compression, the triangular units within the RRH honeycomb contract inward, resulting in an overall inward deformation trend and providing higher resistance stress at larger re-entrant angles; in contrast, the LRRH honeycomb incorporates internal ribs, enabling the parallelogram units to resist axial loads and transfer forces to the external hexagonal units, thus exhibiting an overall positive Poisson’s ratio effect. Due to this alteration in the deformation mechanism, the LRRH structure becomes less capable of achieving rapid densification during the initial compression stage when the re-entrant angle is large. Consequently, the energy absorption process is slower, and energy absorption efficiency is significantly reduced during the later stages of compression. Quantitatively, the SEA of the RRH-Type I-180°-180° structure is 121.2% and 11.79% higher than that of the LRRH-Type I-180°-180° and LRRH-Type I-105°-105° structures, respectively. Meanwhile, the SEA of the LRRH-Type I-105°-105° structure shows a 28.44% improvement over that of the RRH-Type I-105°-105° structure. These findings suggest that while transforming a parallelogram-based structure into a regular triangular re-entrant configuration increases the overall structural mass, it does not necessarily enhance stability and energy absorption capacity. On the contrary, such a transformation may compromise these performance metrics to a certain extent.

### 4.2. The Design and Research of Symmetrical Structures

**Figure 17:**
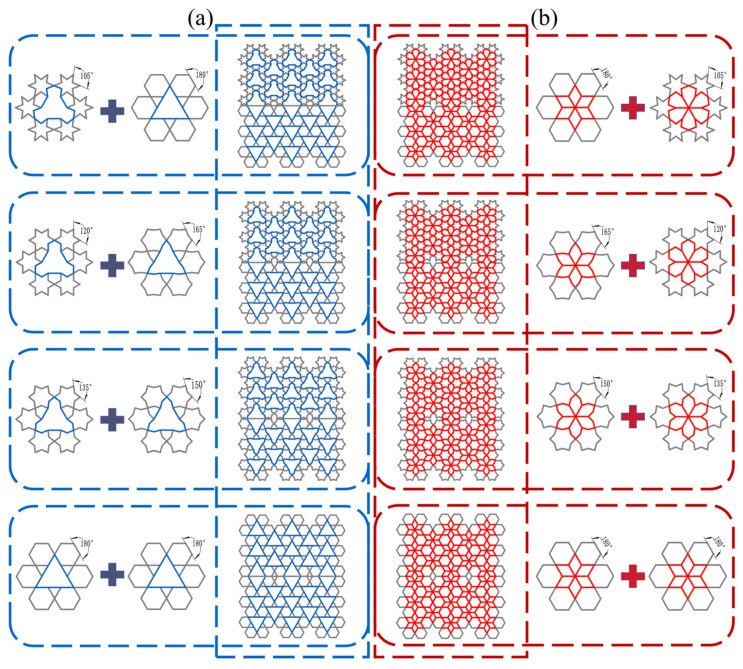
Symmetric structure design process: (**a**) RRH-Type II; (**b**) LRRH-Type II.

Based on the preceding analysis, the RRH and LRRH structures exhibit divergent trends in mechanical performance with increasing concave angle; however, neither configuration achieves optimal energy absorption or load-bearing capacity. To further investigate the influence of re-entrant angle on the mechanical behavior of honeycomb structures, a novel, symmetric honeycomb configuration was designed and is presented in this subsection, as illustrated in Figure 17. The initial design concept was inspired by a symmetric honeycomb unit with a re-entrant angle of 180°, to which multiple units with varying re-entrant angles were subsequently incorporated. These units were then randomly assembled to create the new structure, as depicted in Figure 17a,b, where RRH-Type II and LRRH-Type II represent typical symmetric honeycomb structure assemblies. Throughout the investigation, the axial impact velocity was consistently maintained at 10 m/s, and the wall thickness of each honeycomb unit was established at 0.8 mm. Given the slight variation in the overall height of the honeycomb structures arising from the symmetric configuration, the compression distance was uniformly defined as 99.768 mm, corresponding to a compression ratio of approximately 78.5–80%, to ensure a consistent basis for comparative analysis across different models under identical compression conditions.

**Figure 18:**
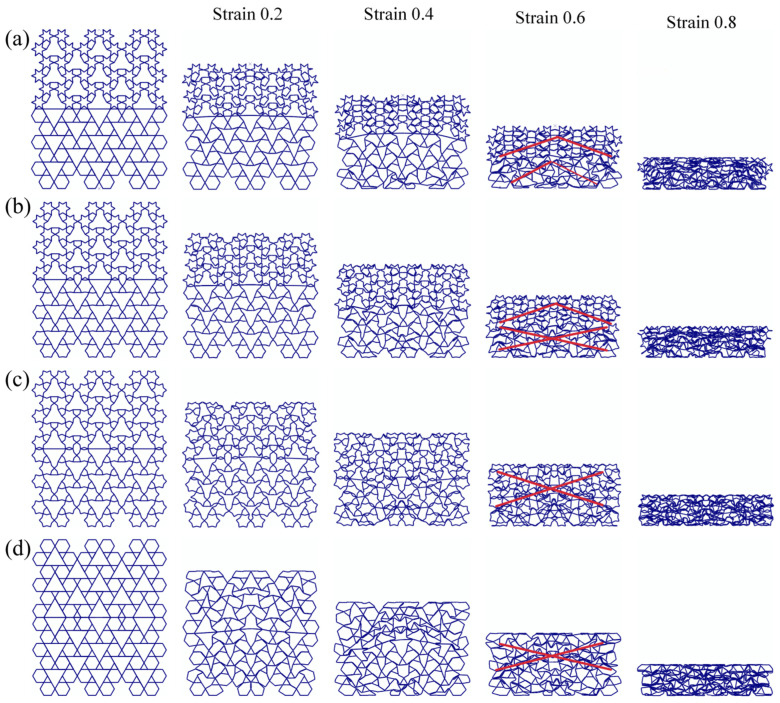
Deformation modes of RRH-Type II structures: (**a**) RRH-Type II-105°-180°; (**b**) RRH-Type II-120°-165°; (**c**) RRH-Type II-135°-150°; (**d**) RRH-Type II-180°-180°.

Figure 18 presents the geometric schematic and deformation patterns of the RRH-Type II honeycomb structures. At a nominal strain of 0.6, distinct local deformation characteristics are observed across various structural configurations. Specifically, the RRH-Type II-105°-180° honeycomb demonstrates pronounced inward “V”-shaped collapse at both the top and bottom during compression. In contrast, the RRH-Type II-120°-165° structure displays a composite deformation mode characterized by a “V”-shaped collapse at the top and an “X”-shaped collapse at the bottom. The RRH-Type II-135°-150° and RRH-Type II-180°-180° configurations predominantly exhibit a symmetric “X”-shaped deformation pattern. These deformation modes indicate that RRH-Type II honeycomb structures undergo significant inward contraction under axial loading, wherein the triangular support ribs experience plastic bending as a result of internal squeezing, thereby effectively bearing and dissipating the external impact load. Furthermore, when there is a notable difference between the top and bottom concave angles, the deformation tends to evolve independently at both ends, collapsing according to their inherent modes. Conversely, when the top and bottom angles are similar, the structure displays a more coordinated and unified deformation behavior.

**Figure 20:**
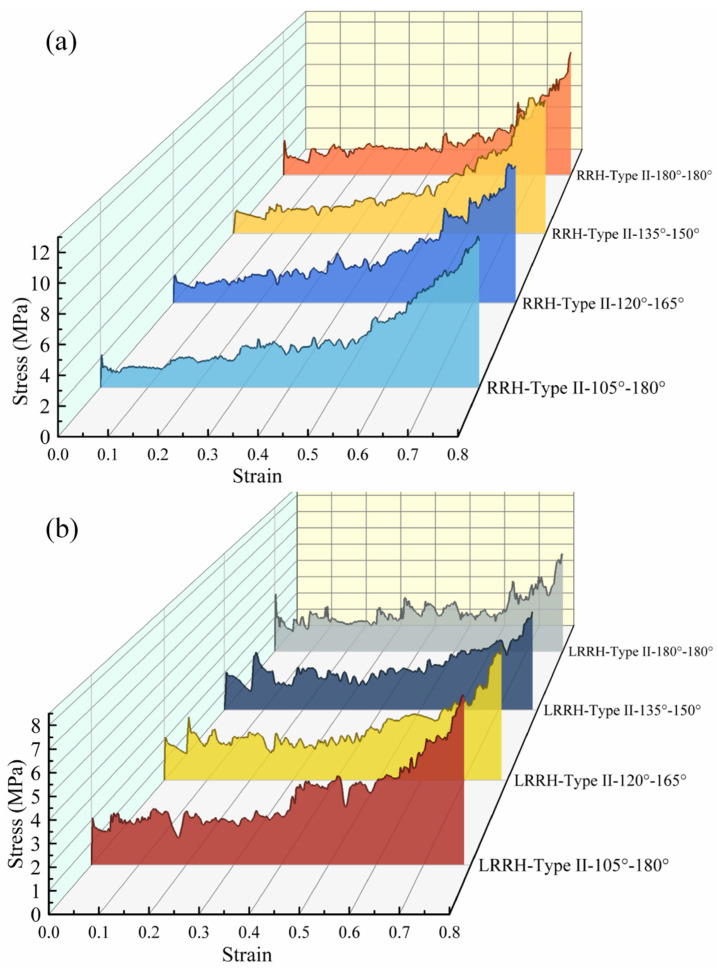
Stress–strain curves: (**a**) RRH-Type II; (**b**) LRRH-Type II.

**Figure 21:**
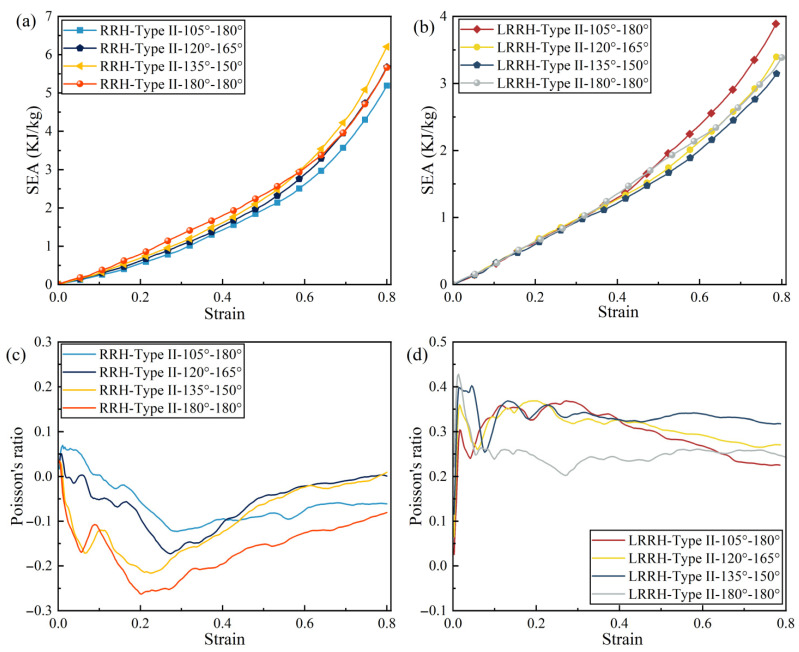
SEA curves and Poisson’s ratio curves for symmetric structures: (**a**) RRH-Type II SEA; (**b**) LRRH-Type II SEA; (**c**) RRH-Type II dynamic Poisson’s ratio; (**d**) LRRH-Type II dynamic Poisson’s ratio.

**Figure 22:**
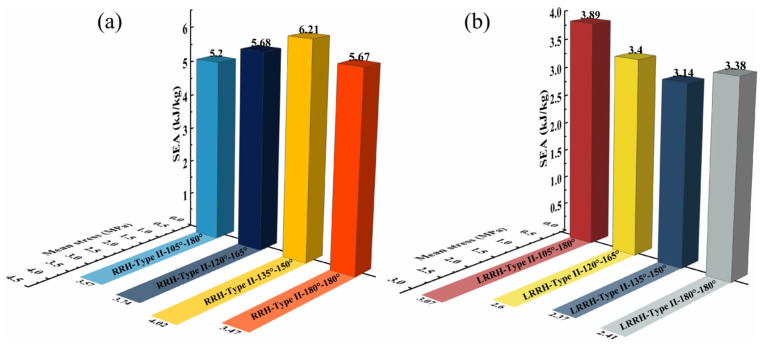
Performance comparison of symmetric structures: (**a**) RRH-Type II; (**b**) LRRH-Type II.

Figure 20a illustrates the stress–strain curves of the RRH-Type II honeycomb structures, where the initial peak stress exhibits a trend of first decreasing and then increasing. An examination of the SEA curves in Figure 21a reveals that prior to a nominal strain of 0.58, the SEA of the RRH-Type III-180°-180° honeycomb consistently exceeds that of the other configurations. This observation indicates that this structure demonstrates superior stability and energy absorption capacity during the early stages of compression. The primary reason for this lies in its pronounced inward contraction behavior at the onset of compression, which effectively resists axial impact loads, resulting in relatively minimal longitudinal deformation and enhanced structural stiffness—thereby improving energy absorption efficiency in the initial stage. In contrast, other symmetric structures tend to undergo longitudinal compression in the upper portion of the honeycomb during the early phase, which inhibits the full development of plastic deformation and leads to lower energy absorption efficiency. As the compression process progresses, the RRH-Type II-180°-180° honeycomb structure gradually exhibits localized collapse in its upper region, leading to discontinuities in internal stress transmission paths and a reduction in stress concentration. Consequently, the growth rate of its SEA slows down or even declines. In contrast, the RRH-Type II-135°-150° honeycomb structure demonstrates a more stable and coordinated global deformation mode, effectively activating a greater number of supporting ribs to undergo plastic deformation. During the later stages of compression, its SEA gradually surpasses that of the RRH-Type II-180°-180° structure. As illustrated in the dynamic Poisson’s ratio curve in Figure 21c, the RRH-Type II-180°-180° honeycomb exhibits the most pronounced negative Poisson’s ratio effect, characterized by a significantly negative value. Specifically, the RRH-Type II-135°-150° structure reaches a Poisson’s ratio of –0.22, indicating that it also exhibits a certain degree of negative Poisson’s ratio behavior during deformation. As shown in Figure 22a, the RRH-Type II-135°-150° honeycomb structure outperforms other symmetric configurations in terms of both SEA and $\sigma_{m}$. Compared with the RRH-Type II-180°-180° structure, its SEA increases by 9.52%, and its $\sigma_{m}$ improves by 15.8%. This enhancement in performance is mainly ascribed to the effective activation of a greater number of triangular supporting ribs undergoing plastic bending, which helps to prevent stress imbalance caused by localized collapse. In summary, the RRH-Type II-135°-150° honeycomb structure achieves a favorable synergistic effect between load-bearing capacity and energy absorption performance, demonstrating superior energy absorption efficiency and structural stability.

**Figure 19:**
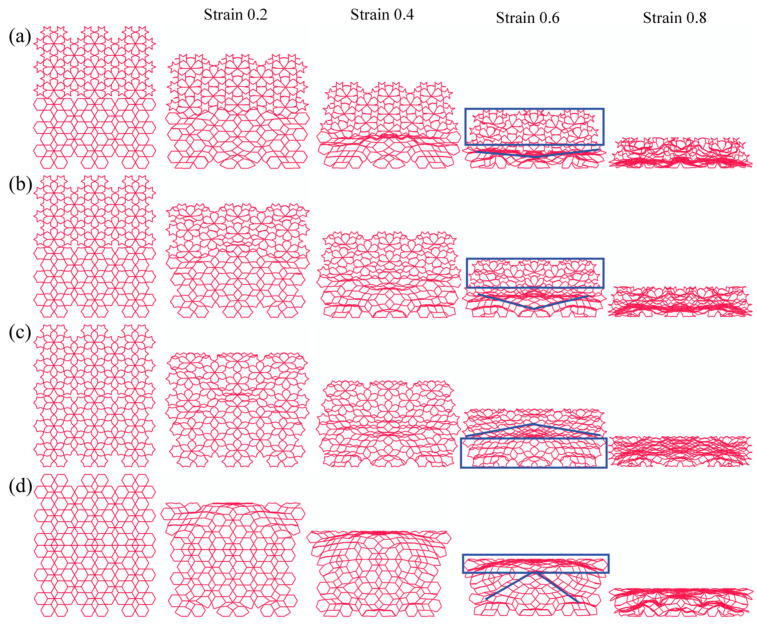
Deformation modes of LRRH-Type II structures: (**a**) LRRH-Type II-105°-180°; (**b**) LRRH-Type II-120°-165°; (**c**) LRRH-Type II-135°-165°; (**d**) LRRH-Type II-180°-180°.

Figure 19 illustrates the geometric configuration and deformation patterns of the LRRH-Type II honeycomb structures. Upon reaching a nominal strain of 0.6, all symmetric configurations exhibit an “I”-shaped deformation mode, characterized by a globally uniform downward compression. Specifically, the LRRH-Type II-105°-180°, LRRH-Type II-120°-165°, and LRRH-Type II-180°-180° structures demonstrate an “I”-shaped compression pattern in the upper region, which gradually transitions into a “V”-shaped contraction in the lower region, forming a distinct upper–lower deformation zoning. In contrast, the LRRH-Type II-135°-150° structure exhibits the opposite deformation behavior, with a “V”-shaped contraction occurring in the upper part, while the lower part maintains a relatively regular “I”-shaped compression. These differences primarily arise from the substantial impact of concave angle parameters on the distortion mechanisms of internal supporting ribs: when the concave angle is larger, the internal ribs are more prone to buckling and plastic deformation, leading to pronounced local contraction and the formation of “V”-shaped zones; conversely, smaller concave angles enhance overall structural stability, resulting in more uniform compression and less localized deformation, manifesting as a consistent “I”-shaped downward compression.

Figure 20b depicts the stress–strain curves of the LRRH-Type II honeycomb structures. The variation trend of the initial peak stress is generally consistent with that of the RRH-Type II series, exhibiting a decrease followed by an increase. Notably, the LRRH-Type II-105°-180° structure encompasses the largest area with respect to the *X*-axis. Combined with the SEA curves shown in Figure 21b, it is observed that when the nominal strain is below 0.44, the SEA values of different structures are relatively similar. However, as the compression deepens and the strain exceeds 0.44, the SEA of the LRRH-Type II-105°-180° structure increases rapidly, significantly outperforming other symmetric configurations. This behavior is primarily ascribed to the distortion characteristics of the structure under compression. In the early compression stage, segments with larger concave angles exhibit greater flexibility, causing the internal support ribs to undergo local bending and buckling, thereby providing an effective cushioning effect. Meanwhile, segments with smaller concave angles possess higher structural stiffness and gradually engage in load-bearing during the later compression stages, providing sustained compressive resistance. As shown in Figure 21d, the LRRH-Type II series consistently exhibits a positive Poisson’s ratio effect. This indicates that despite the symmetric combination strategy, the Poisson’s ratio behavior of the structure does not undergo significant changes. However, by adjusting the internal geometry of the honeycomb units, the Poisson’s ratio effect can be effectively tuned. In fact, the emergence of negative Poisson’s ratio behavior is closely related to the internal geometric features of the structure. Thus, symmetric combinations alone are insufficient to induce auxetic behavior. Rather, optimizing the geometry of individual honeycomb cells—particularly the layout and structural characteristics of the internal elements—is crucial to achieving negative Poisson’s ratio characteristics. Further evidence is provided in Figure 22b, which shows that the LRRH-Type II-105°-180° honeycomb outperforms other symmetric configurations in both SEA and $\sigma_{m}$. Compared with the LRRH-Type II-180°-180° structure, its SEA increases by 15.1%, and $\sigma_{m}$ is improved by 27.4%. This significant performance advantage suggests that the LRRH-Type II-105°-180° honeycomb exhibits excellent energy absorption potential under axial impact loads, effectively dispersing energy while maintaining structural integrity.

In summary, the differences in mechanical performance between the RRH-Type II and LRRH-Type II symmetric honeycomb structures are primarily attributed to the influence of internal re-entrant angles on the deformation behavior of the supporting ribs. The RRH-Type II structures achieve negative Poisson’s ratio behavior and stable early-stage energy absorption through coordinated inward contraction and plastic bending of the triangular support ribs, whereas the LRRH-Type II structures rely on the staged activation induced by differences in re-entrant angles, exhibiting positive Poisson’s ratio behavior and achieving efficient energy dissipation through a sequential “soft-to-stiff” load-bearing mechanism.

## 5. Parameterization Analysis

### 5.1. Influence of Wall Thickness

**Figure 23:**
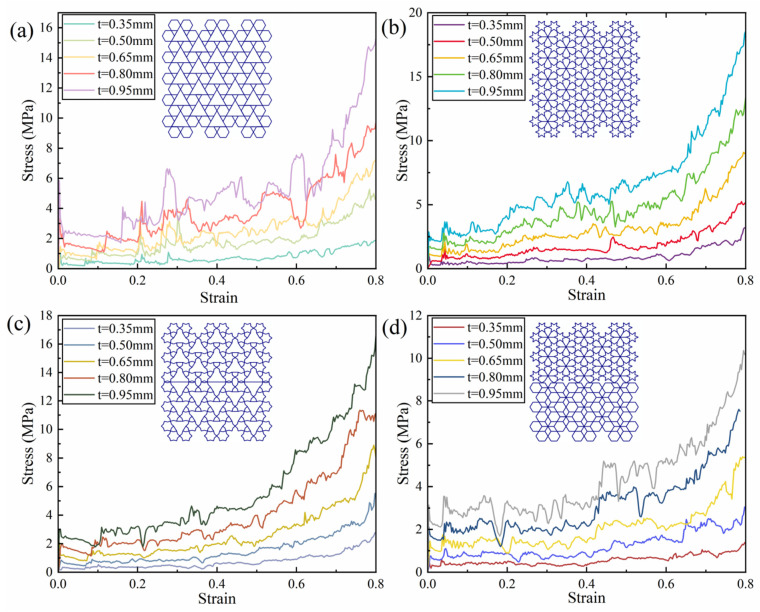
Stress–strain curves of structures with different wall thicknesses: (**a**) RRH-Type II-180°-180°, (**b**) LRRH-Type II-105°-105°, (**c**) LRRH-Type II-135°-150°, and (**d**) LRRH-Type II-105°-180°.

The wall thickness of honeycomb structures exerts a significant influence on their mechanical performance. This section is dedicated to examining the effects of wall thickness variations on the response of honeycomb structures subjected to axial impact loading. Four representative configurations—RRH-Type I-180°-180°, LRRH-Type I-105°-105°, RRH-Type II-135°-150°, and LRRH-Type II-105°-105°—were selected for analysis. Dynamic analysis was carried out at a collision velocity of 10 m/s, considering five different wall thicknesses (0.35 mm, 0.5 mm, 0.65 mm, 0.8 mm, and 0.95 mm). As illustrated in Figure 23, the stress–strain curves under varying wall thicknesses reveal a distinct trend: the plateau stress demonstrates a marked increase with increasing wall thickness, and the onset of densification is observed to occur earlier. This behavior can be primarily attributed to the enhancement of bending stiffness in the cell walls, which enables more effective resistance to local buckling and unstable deformation during compression, thereby preserving structural integrity. The underlying mechanisms responsible for this phenomenon can be elucidated as follows: First, the augmentation of wall thickness significantly enhances the bending stiffness of the honeycomb cell walls, enabling them to more effectively resist local buckling and unstable deformation during compression, thus maintaining structural integrity. Second, greater wall thickness reduces the porosity between adjacent cells, leading the honeycomb structure to advance into the densification phase sooner. During this process, intercellular contact becomes more intensive, allowing for rapid compaction of the internal structure. This results in a denser load transfer path, which substantially boosts the structure’s load-carrying capability and stress response.

**Figure 24:**
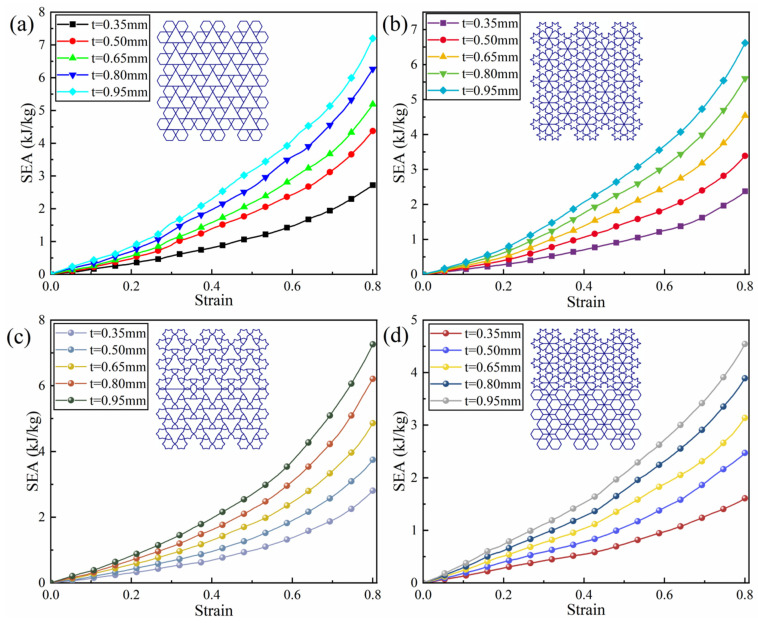
Specific energy absorption curves of structures with varying wall thicknesses: (**a**) RRH-Type I-180°-180°, (**b**) LRRH-Type I-105°-105°, (**c**) LRRH-Type II-135°-150°, and (**d**) LRRH-Type II-105°-180°.

Figure 24 presents the SEA–strain curves, which clearly demonstrate that the SEA increases significantly with wall thickness. According to the data in Table 2, when the wall thickness increases from 0.35 mm to 0.95 mm, the SEA of the RRH-Type I-180°-180° structure increases by 164.7%, that of the LRRH-Type I-105°-105° structure increases by 178.2%, the SEA of the RRH-Type II-135°-150° structure rises by 158.4%, and the SEA of the LRRH-Type II-105°-105° structure shows a remarkable 182% improvement. Overall, the SEA values of these structures increase by approximately 1.5 to 2 times. The elevated SEA is primarily credited to the heightened stiffness and strength of the honeycomb structures consequent upon the increased wall thickness. Thicker cell walls provide greater resistance to bending and buckling under external impact or compressive loads, effectively suppressing local instabilities. This ensures that the structures maintain better geometric integrity during deformation. A stable deformation path facilitates the dissipation of more external energy through material plastic deformation and structural densification, thereby significantly improving the specific energy absorption performance. Table 2 summarizes the comparative performance of RRH and LRRH structures under varying wall thicknesses.

### 5.2. Effect of Impact Velocity

**Figure 25:**
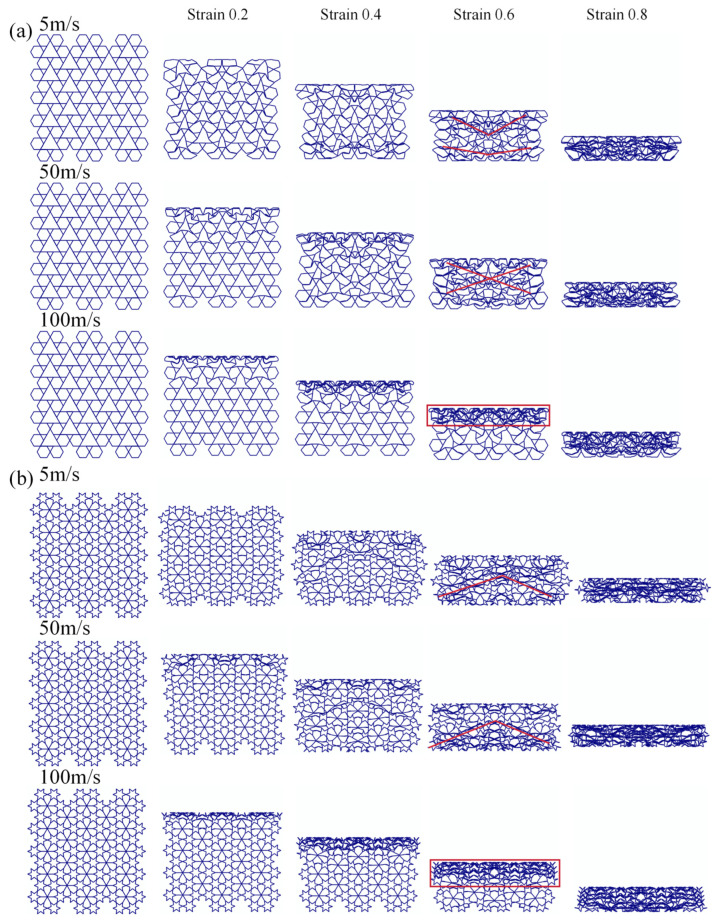
Deformation process of Type I structures under different impact velocities: (**a**) RRH-Type I-180°-180°; (**b**) LRRH-Type I-105°-105°.

**Figure 26:**
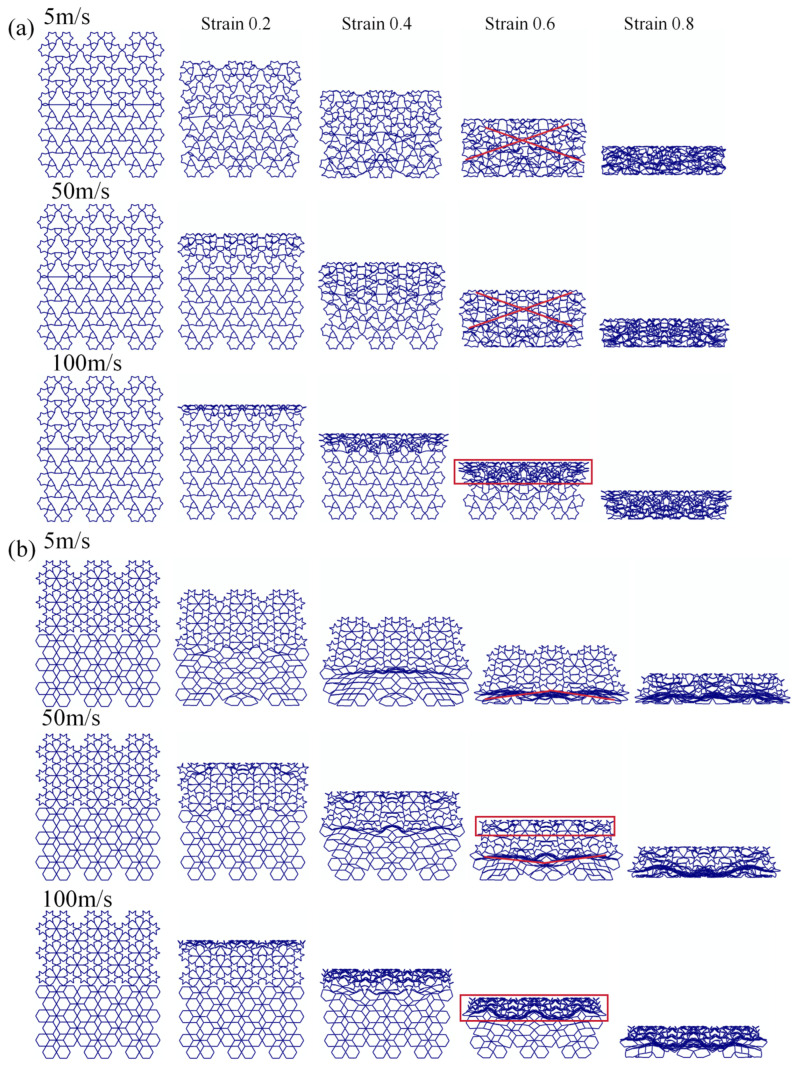
Deformation process of Type II structures under different impact velocities: (**a**) RRH-Type II-135°-150°; (**b**) LRRH-Type I-105°-180°.

The impact velocity has a significant influence on the mechanical response of honeycomb structures. In this section, four representative honeycomb configurations—RRH-Type I-180°-180°, LRRH-Type I-105°-105°, RRH-Type II-135°-150°, and LRRH-Type II-105°-105°—are which are subjected to axial compression simulations at low (5 m/s), medium (50 m/s), and high (100 m/s) impact velocities, while maintaining a constant wall thickness of t = 0.8 mm. The primary objective of this analysis is to examine the effects of impact velocity on deformation patterns and energy absorption performance. Figure 25 and Figure 26 depict the deformation morphologies of the structures under varying impact velocities. At a nominal strain of 0.6, the RRH-Type I-180°-180° honeycomb structure exhibits a “V”-shaped deformation at both the top and bottom under a low-speed impact of 5 m/s. As the impact speed increases to 50 m/s, the deformation transitions to an overall “X”-shaped collapse. Conversely, the LRRH-Type II-105°-105° structure demonstrates a “V”-shaped collapse under the 5 m/s impact; however, as the impact velocity rises to 50 m/s, the upper region undergoes an “I”-shaped overall collapse, while the lower region retains the “V”-shaped characteristic, resulting in a marked asymmetric mixed deformation mode. The LRRH-Type I-105°-105° honeycomb structure maintains a stable “V”-shaped deformation across both the 5 m/s and 50 m/s impact conditions. Similarly, the RRH-Type II-135°-150° structure consistently exhibits a typical “X”-shaped coordinated collapse under both low and medium impact velocities. Upon further increasing the impact velocity to 100 m/s, all four honeycomb structures demonstrate a distinct “I”-shaped overall crushing deformation, indicating a transition toward a rigidity-dominated longitudinal compression mode under high-speed impacts. This evolution of deformation patterns underscores the sensitivity of honeycomb structures to varying loading rates. Under low-speed compression, energy absorption predominantly occurs through localized folding and rib bending, resulting in a relatively progressive and mild deformation process. In contrast, under medium and high-speed impacts, the internal stress transmission paths of the structures undergo significant alterations, leading to simultaneous buckling and collapse of multiple cells and initiating a synergistic deformation effect.

**Figure 27:**
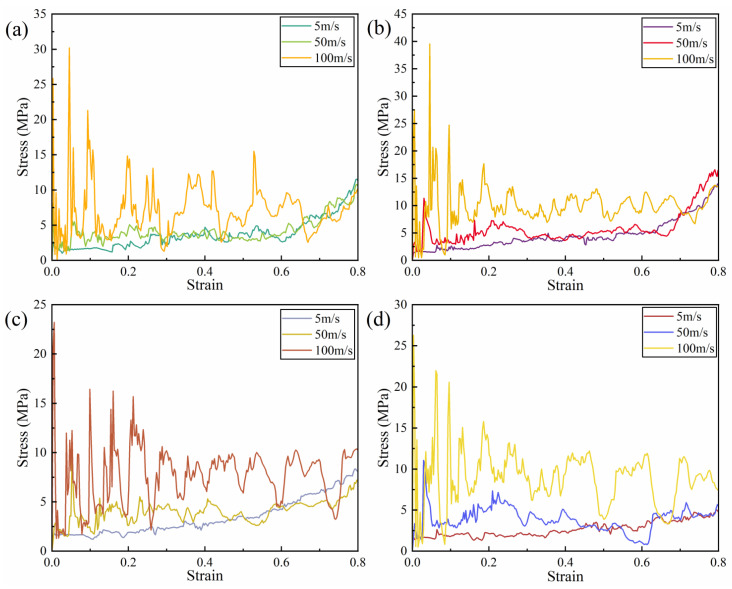
Stress–strain curves at different impact velocities: (**a**) RRH-Type I-180°-180°, (**b**) LRRH-Type I-105°-105°, (**c**) LRRH-Type II-135°-150°, and (**d**) LRRH-Type II-105°-180°.

**Figure 28:**
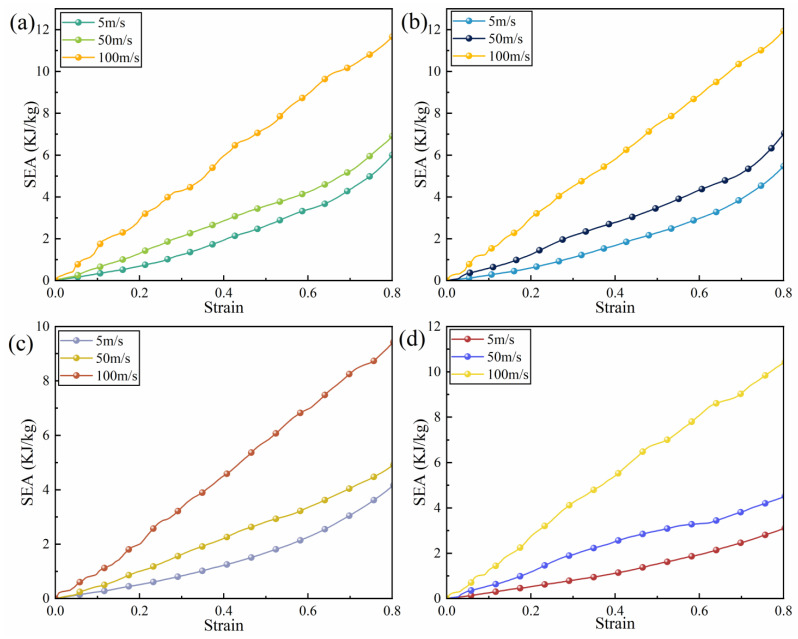
Specific energy absorption (SEA) curves under different impact velocities: (**a**) RRH-Type I-180°-180°; (**b**) LRRH-Type I-105°-105°; (**c**) LRRH-Type II-135°-150°; (**d**) LRRH-Type II-105°-180°.

Figure 27 illustrates the stress–strain curves of the four honeycomb structures under different impact velocities. It is evident that under high-speed impact conditions, the curves exhibit pronounced fluctuations, whereas under low and medium speeds, the curves display partial overlap, indicating a higher degree of consistency and stability. This phenomenon suggests that high-speed loading significantly amplifies inertial effects, resulting in more intense stress transmission within the honeycomb structures. Consequently, localized regions are more susceptible to rapid buckling and instability, which produces noticeable stress oscillations. In contrast, under low and medium impact velocities, the deformation process of the honeycomb structures remains relatively stable, with the supporting ribs undergoing gradual bending and buckling, resulting in a more uniform energy dissipation process and smaller amplitude fluctuations in the stress curves. Figure 28 presents the SEA–strain curves, revealing that SEA values under high-speed impact are substantially elevated compared to those under low and medium-speed impacts. According to the data presented in Table 3, the SEA under high-speed conditions is approximately 2 to 2.9 times greater than that under low-speed impact. Notably, at high impact velocities, the differences in specific energy absorption among the four structures are relatively minor. However, under low-speed impact, the SEA of the RRH-Type I-180°-180° honeycomb structure is 60.86% greater than that of the LRRH-Type II-105°-105° structure. Overall, impact velocity not only influences the stress response characteristics of honeycomb structures but also profoundly affects their internal deformation mechanisms and energy dissipation modes. Table 3 provides a comparative analysis of the mechanical performance of RRH and LRRH structures under varying impact velocities.

## 6. Conclusions

Inspired by rotationally rigid structures, this study proposes a novel rotational hexagonal honeycomb structure by geometrically integrating triangular and hexagonal units through a rotational configuration approach. To further enrich the geometric characteristics, locally re-entrant quadrilateral cells are introduced into the design. The accuracy of the constructed finite element model is validated through quasi-static compression experiments. Based on this, the mechanical response and energy absorption performance of the proposed structure under axial impact loading are systematically investigated.

1. This study first conducted a comparative analysis of the mechanical responses of RRH-Type I and LRRH-Type I honeycomb structures under axial impact loads with varying concave angles. The results show that the SEA (specific energy absorption) of RRH-Type-180°-180° increased by 43.68% compared to RRH-Type-105°-105°; LRRH-Type-105°-105° exhibited a 97.88% higher SEA than LRRH-Type-180°-180°; and RRH-Type-180°-180° outperformed LRRH-Type-105°-105° by 11.79% in SEA. With the increase in concave angles, the two types of structures exhibited distinct evolutionary trends in energy absorption performance. Although the introduction of quadrilateral cells can alter the deformation mode to some extent, this embedded configuration did not demonstrate superior energy absorption capacity or structural stability compared to the original regular triangular configuration.
2. To further advance the design concept of lightweight structures, this study introduces a symmetrical configuration strategy by combining RRH-Type I and LRRH-Type I honeycomb structures to develop RRH-Type II and LRRH-Type II configurations. A systematic comparative analysis of their mechanical performance under axial impact loading was conducted. The results indicate that the SEA (specific energy absorption) of RRH-Type II-135°-150° increased by 9.52% compared to RRH-Type II-185°-180°, while LRRH-Type II-105°-180° exhibited a 15.1% improvement in SEA relative to LRRH-Type II-180°-180°. The performance enhancement of the RRH-Type II structure is primarily attributed to the effective activation of plastic bending in the triangular support ribs, whereas the improved energy absorption of the LRRH-Type II structure results from the effective buffering effect provided by the internal support ribs during impact loading.
3. For the four honeycomb structures with superior energy absorption performance mentioned above, the influence of wall thickness on their mechanical behavior was further investigated. The results indicate that increasing the unit wall thickness significantly enhances the structure’s resistance to bending and buckling under external impact or compressive loading, effectively suppressing local instabilities and enabling the structure to maintain favorable deformation patterns and overall structural integrity. The increase in wall thickness also leads to a substantial improvement in stiffness and strength, thereby further enhancing the energy absorption capacity. These findings provide a viable and effective strategy for optimizing the energy-absorbing performance of honeycomb structures.
4. Building upon the four honeycomb structures with superior energy absorption performance, the influence of impact velocity on their mechanical response was further analyzed. The results demonstrate that under high-velocity impact, all structures exhibit a characteristic “I”-shaped collapse deformation mode. During this process, the significantly intensified inertial effects lead to more violent internal stress transmission, making local regions more susceptible to rapid buckling and instability, which in turn triggers pronounced stress fluctuations. In contrast, under low to moderate impact velocities, the deformation process is relatively stable; the supporting ribs undergo gradual bending and buckling, resulting in a more uniform energy dissipation process. Consequently, the stress response becomes smoother, with substantially reduced fluctuation amplitudes.
5. By introducing rotating rigid elements and combining them with a ribbing design, the internal load transfer of the structure is optimized, and the local constraints are strengthened, thereby achieving stable and progressive folding deformation during compression, effectively suppressing local instability and enhancing the platform stage’s load-bearing stability and overall energy absorption efficiency.

## Figures and Tables

**Table 1:** Material properties of PLA.

ρ (kg/m^3^)	E (MPa)	$\sigma_{y}$ (Mpa)	ν
$1.25\times 10^{3}$	2751	42.1	0.35

**Table 2:** Comparative performance of RRH and LRRH structures with different wall thicknesses.

Honeycombs	t (mm)	V (m/s)	m (g)	EA (J)	SEA (KJ/Kg)
RRH-Type I-180°-180°	0.35	10	31.6	86.03	2.72
	0.50	10	49.7	217.38	4.37
	0.65	10	58.8	305.00	5.19
	0.80	10	72.3	452.62	6.26
	0.95	10	85.9	618.31	7.20
LRRH-Type I-105°-105°	0.35	10	43.4	104.10	2.38
	0.50	10	6.19	209.82	3.39
	0.65	10	80.5	365.68	4.54
	0.80	10	99.1	555.13	5.60
	0.95	10	117.7	779.17	6.62
RRH-Type II-135°-150°	0.35	10	33.6	94.35	2.81
	0.50	10	52.3	195.96	3.75
	0.65	10	68.0	330.35	4.86
	0.80	10	83.7	519.57	6.21
	0.95	10	99.4	721.77	7.26
LRRH-Type II-105°-180°	0.35	10	44.8	72.12	1.61
	0.50	10	63.9	157.96	2.47
	0.65	10	83.1	260.60	3.14
	0.80	10	102.0	396.84	3.90
	0.95	10	121.5	552.04	4.54

**Table 3:** Performance comparison of RRH and LRRH structures under different impact velocities.

Honeycombs	t (mm)	V (m/s)	m (g)	EA (J)	SEA (KJ/Kg)
RRH-Type I-180°-180°	0.80	5	72.3	433.77	6.00
	0.80	50	72.3	499.13	6.90
	0.80	100	72.3	842.96	11.66
LRRH-Type I-105°-105°	0.80	5	99.1	542.18	5.47
	0.80	50	99.1	769.73	7.77
	0.80	100	99.1	1182.87	11.94
RRH-Type II-135°-150°	0.80	5	83.7	503.05	5.16
	0.80	50	83.7	587.21	6.03
	0.80	100	83.7	1017.07	10.44
LRRH-Type II-105°-180°	0.80	5	102.0	380.35	3.73
	0.80	50	102.0	529.69	5.19
	0.80	100	102.0	1107.37	10.86

## Data Availability

The original contributions presented in this study are included in the article. Further inquiries can be directed to the corresponding author.
